# Commencement of the Baltimore College of Dental Surgery

**Published:** 1848-04

**Authors:** 


*Commencement of the Baltimore College of Dental Surgery.*—The annual commencement of the Baltimore College of Dental Surgery, for conferring degrees, was held in the saloon of the college building, on Thursday evening, March 2nd, and notwithstanding the inclemency of the night, a very good audience was in attendance to witness the ceremony.

After music by the band, the candidates for the honors of the institution were called up by professor Harris, and the degree of Doctor of Dental Surgery conferred by professor Bond, on the following gentlemen, viz. Daniel Vandinburg, N. Y., R. W. Armstrong, Md., John M'Calla, Pa., B. A. Kennedy, N. C., Chas. Bond, Md., R. D. Addington, Va., W. H. Morgan, Ky., Joshua King, N. C., Thos. J. Jones, Ga., Hervey Colburn, M. D. Md., E. W. Mason, Md., Charles Barnes, Md. D. G. Varney, Mass., and J. J. Adair, Ky.

After this ceremony was completed, and music again, Dr. E. Parmly, of N. Y., arose and pronounced the Valedictory Address. He referred to the difficulties that the institution had in its infancy to struggle with, but which had all vanished before the energetic and persevering efforts that supported it, and now presented itself as the chief agent of professional elevation, standing upon a basis not to be shaken by the envious and sneering. He said that it was to this institution that the better portion of the profession turned their attention and expectations for the cultivation, growth, and respectability of the profession. That part of his address that particularly referred to the graduating class, was marked by wisdom and feeling.

Dr. J. J. Adair replied to Dr. Parmly on behalf of the graduating class, in a manner highly complimentary to himself, and expressive of the sentiments of those whom he represented. He thanked Dr. Parmly for the expression of interest in the Alumni of the institution, and particularly to the faculty for the manner with which they had labored to instruct them in the knowledge of their profession. Professor Bond made a brief reply from the faculty, expressing feelings of great gratification for the sentiments expressed by Dr. Adair.

The Awarding Committee then made their report through Dr. Gardette, who announced the name of Dr. Vandinburg, of New York, as the candidate that had been selected, after two days examination by the committee, to receive the award of a beautiful set of extracting instruments.

After music by the band, the class, with a few friends, retired to an entertainment served up in one of the rooms of the building.

We are in hopes to be put in possession of the addresses delivered on the occasion, so as to present them to our readers.—*Dental Intelligencer.*

